# Identification of essential proteins based on edge features and the fusion of multiple-source biological information

**DOI:** 10.1186/s12859-023-05315-y

**Published:** 2023-05-17

**Authors:** Peiqiang Liu, Chang Liu, Yanyan Mao, Junhong Guo, Fanshu Liu, Wangmin Cai, Feng Zhao

**Affiliations:** 1 School of Computer Science and Technology, Shandong Technology and Business University, Yantai, China; 2 College of Oceanography and Space Informatics, China University of Petroleum (East China), Qingdao, China

**Keywords:** Essential protein, Quasi-clique, Triangle graph, Dynamic protein–protein interaction network, Fusion method

## Abstract

**Background:**

A major current focus in the analysis of protein–protein interaction (PPI) data is how to identify essential proteins. As massive PPI data are available, this warrants the design of efficient computing methods for identifying essential proteins. Previous studies have achieved considerable performance. However, as a consequence of the features of high noise and structural complexity in PPIs, it is still a challenge to further upgrade the performance of the identification methods.

**Methods:**

This paper proposes an identification method, named CTF, which identifies essential proteins based on edge features including *h*-quasi-cliques and *uv*-triangle graphs and the fusion of multiple-source information. We first design an edge-weight function, named EWCT, for computing the topological scores of proteins based on quasi-cliques and triangle graphs. Then, we generate an edge-weighted PPI network using EWCT and dynamic PPI data. Finally, we compute the essentiality of proteins by the fusion of topological scores and three scores of biological information.

**Results:**

We evaluated the performance of the CTF method by comparison with 16 other methods, such as MON, PeC, TEGS, and LBCC, the experiment results on three datasets of *Saccharomyces cerevisiae* show that CTF outperforms the state-of-the-art methods. Moreover, our method indicates that the fusion of other biological information is beneficial to improve the accuracy of identification.

## Background

Proteins are the material basis of life activities. They can be divided into essential and non-essential proteins. The cell becomes nonfunctional or dysfunctional when essential proteins are knocked out [1]. Identification of essential proteins can help us uncover the mechanisms of cell aging and aging-related diseases and is of great significance to disease diagnosis and drug design [2].

Essential proteins have been identified by biological experimental approaches and computing methods. The advantage of biological experimental methods, such as gene knockout, conditional knockout, and RNA interference [3], is high reliability, but the disadvantages are that they are time-consuming and expensive [4]. With the rapid development of high-throughput experimental methods, protein–protein interaction (PPI) data have been enriched. Consequently, it is possible to identify essential proteins using computing methods [5].

Interactions among proteins can be modeled by a simple graph where a vertex corresponds to a protein and an edge to an interaction, also called a protein–protein interaction network (PIN). In a PIN, highly connected vertices tend to be essential based on the centrality–lethality rule proposed by Jeong et al [6]. Accordingly, computing methods identify essential proteins by the topological features of PINs [7]. For these methods, centrality measures are crucial. Much research in recent years has focused on centrality measures, such as degree centrality (DC) [8], betweenness centrality (BC) [9], closeness centrality (CC) [10], subgraph centrality (SC) [11], eigenvector centrality (EC) [12], information centrality (IC) [13], local average centrality (LAC) [14], and neighbor centrality (NC) [15]. It must be also mentioned that previous research shows that we cannot identify all essential proteins based on existing centrality measures, because of noise in PINs, limitations of centrality measures, and other reasons [16]. It remains challenging to develop novel centrality measures to further improve the performance of the identification methods [17].

Besides centrality measures, previous research shows that it is helpful for identifying essential proteins to fuse multisource biological information [18], such as GO annotations, protein complexes, gene expression profiles, and subcellular localization. Fusion methods can be generally grouped into three categories: edge weight methods, PIN reconstruction methods, and fusion methods.

The basic idea of edge weight methods is to identify essential proteins via an edge-weighted PIN, whose edges are weighted based on topological features and biological information. Edge-weighted PINs can be obtained via the fusion of gene expression profiles, such as the methods proposed by Tang et al. (WDC) [19], Zhang et al. (CoEWC) [20], Li et al. (PeC) [21], and Zhong et al. (JDC) [22]. GO annotations are another kind of biological information used to assign a weight to an edge [23], for example, the method GEG presented by Zhang et al. [24]. Previous studies have demonstrated that the number of protein domain types contained in a protein is highly correlated with its essentiality, for example, the model NPRI developed by Chen et al. [25]. Based on the relation between the orthology and essential proteins, Peng et al. proposed the method ION [26]. Recently, to further enhance accuracy, some methods generate an edge-weighted PIN by simultaneously fusing several kinds of biological information, such as esPOS [27], TEO [28], and TEGS [29].

To decrease the influence of noise or incompleteness inherently existing in PINs, the key point of PIN reconstruction methods is to reconstruct a PIN using biological information. In the study of Wang et al., a dynamic PIN (DPIN), which consists of a series of time-sequenced subnetworks that are static PINs, was constructed by combining gene expression data with PINs for denoising PINs [30]. The WPDINM model proposed by Meng et al. estimates the essentiality of proteins based on subcellular localization, orthologous information, and a novel weighted protein–domain interaction network constructed by PINs and gene expression profiles [31]. On the basis of the relations between protein functions and subcellular localization, Li et al. presented the SPP method [32]. Zhao et al. presented two methods, DSN and MON, by integrating PINs, protein domains, gene expression profiles, orthologous proteins, and subcellular localization information [33, 34].

The fundamental strategy of fusion methods is to identify essential proteins through weighted scores computed using other kinds of biological information or other methods, which are complementary, that is, the essential protein sets identified by these methods are different [18, 21, 35]. By fusion of PINs, orthologous proteins, and subcellular localization, the SON method was presented by Li et al. [36]. The LIDC method proposed by Luo et al. computes weighted scores using PINs and protein complex information [37]. Based on the TEGS method, Zhang et al. proposed the CEGSO method through fusing subcellular locations and two other methods [5], namely, IDC [37] and NOS. Based on the combination of local density, BC and IDC, Qin et al. presented the LBCC method [38].

Although all the previously mentioned identification methods have demonstrated good performance, they suffer some disadvantages, and there is room for enhancement. For concerning the methods based on centrality measures, the limitation is that these measures are not sufficient to perfectly characterize the complete features of essential proteins. There remains a need for efficient centrality measures that can compute the essentiality of lowly or highly connected proteins, because lowly connected proteins may be essential and highly connected proteins maybe not. For example, there are 321 essential proteins whose interactions are less than or equal to 3 and there are 809 non-essential proteins whose interactions are greater than average in the DIP dataset (see Section “Experiments and discussions”), which contains 1167 essential proteins out of 5093 proteins. The example is inconsistent with the assumption that highly connected proteins tend to be essential. Therefore, how to design a method to identify the two types of proteins by deeply analyzing the topological features of PINs is still an important question. For the methods based on fusing multi-source biological information, it is still a challenge to identify more inherent potential relations between essential proteins and biological properties in different kinds of biological information.

To tackle the limitations mentioned above, we present a novel method for identifying essential proteins, named CTF (the identification method of essential proteins based on edge features including *h*-quasi-cliques and *uv*-triangle graphs, and the fusion of multiple-source biological information). To our knowledge, it is the first time that the concepts of *h*-quasi-cliques and *uv*-triangle graphs are considered in the identification of essential proteins. The contributions of this paper are summarized as follows.

1. For constructing an edge-weighted PIN, we propose an function, named EWCT (the edge weight function based on edge features *h*-quasi-cliques and *uv*-triangle graphs by combining with GO annotations), to weight edges.
2. To denoise PINs and further enhance their performance, we construct an edge-weighted PIN using EWCT and a DPIN.
3. To further enhance accuracy, the CTF method computes three essential scores of proteins using three kinds of biological information, namely, protein complexes, subcellular localization, and orthologous information, and the CTF method is upgraded by optimizing the weights of the different essential scores.

To verify the effectiveness and superiority of CTF, we design experiments on three different yeast PINs and compare CTF with 16 methods, including MON, PeC, TEGS, and LBCC. The results show that CTF has higher performance than the other methods.

## Definitions and notations

Let us introduce some notations and terminologies before describing the CTF method in detail. A PIN is typically modeled by a simple graph $G = (V, E)$ with a set of vertices *V* and a set of edges *E*, where vertices and edges represent proteins and interactions, respectively. For an edge $e \in E$ incident on *u* and *v*, denote the edge *e* by $e = (u, v)$ or (*u*, *v*), and we say that *u* and *v* are “adjacent” or *u* is a “neighbor” of *v*. The *k*th-order neighbors of vertex *u* are a set of vertices whose shortest path distances to *u* are equal to *k*, and the *k*th-order nearest neighbors of protein *u* are a set of vertices whose shortest path distances to *u* are less than or equal to *k*. In this paper, for convenience, we interchangeably use the terms “vertex” and “protein” without any confusion because of the one-to-one mapping between the vertex set and the protein set and similarly for “edge” and “interaction”.

In a simple graph $G = (V, E)$, the “degree” of a vertex *u* is the number of edges incident on it. Let *d*(*v*) denote the degree of *v*, and *N*(*v*) denote the set of neighbors of *v*. The union of *N*(*u*) and *N*(*v*), denoted by $N(u) \cup N(v)$, is the set of vertices that are in *N*(*u*) or *N*(*v*) or both *N*(*u*) and *N*(*v*), and the intersection of *N*(*u*) and *N*(*v*), denoted by $N(u) \cap N(v)$, is the set of vertices that are in both *N*(*u*) and *N*(*v*). The set $N(u) \cap N(v)$ is called the common neighbor set of *u* and *v*.

An edge-weighted graph is a graph that has a number, called a weight, associated with each edge. We denote the weight of the edge e incident on vertices *u* and *v* by *w*(*e*(*u*, *v*)).

Given a simple graph $G = (V, E)$, *G* is a clique if *u* is adjacent to *v* for arbitrary two distinct vertices *u* and *v* of *V*. Therefore, given a clique with *n* vertices, it has $(n * (n - 1)) / 2$ edges. The maximal clique problem is to find a clique that is not contained in any other clique in a graph. In real-world contexts, we need to relax a clique problem to an almost-clique problem, that is, dense incomplete graphs, also called quasi-cliques, which generalize the notion of cliques. In our method, we define a variant of cliques: *h*-quasi-cliques.

### Definition 1

(*h*-quasi-clique) For a simple graph *G* with *n* vertices, *G* is an *h*-quasi-clique such that the number of edges in *G* is greater than or equal to $(n * (n - 1)) / 4$, that is, half the number of edges of a clique with *n* vertices.

Given a simple graph $G = (V, E)$, for each $v \in V$, if *G* contains at least one subgraph that is a triangle and contains vertex *v*, we say that *G* is a triangle graph. A variant of a triangle graph is a *uv*-triangle graph.

### Definition 2

(*uv*-triangle graph) Given a simple graph $G = (V, E)$, we say that *G* is a *uv*-triangle graph if it satisfies the *uv*-triangle condition: there exists an edge $e = (u, v)$ for each vertex $w \in V$ such that ${w, w_1, w_2}$ induces a triangle in *G*, where $w_1$ and $w_2 \in {u, v} \cup (N(u) \cap N(v))$. The triangle is called a triangle graphlet of *G*.

**Fig. 1:**
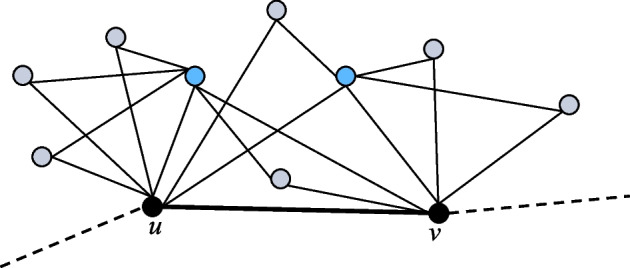
The subgraph $G_{uv}$ induced by solid edges is an *h*-quasi-clique and is also a *uv*-triangle graph

For example, Fig. 1 illustrates a subgraph that is an *h*-quasi-clique and is also a *uv*-triangle graph, where the blue vertices belong to $N(u) \cap N(v)$, and the gray vertices belong to $(N(u) \cup N(v)) - (N(u) \cap N(v))$.

For a graph *G*, if *G* is an *h*-quasi-clique and is also a *uv*-triangle graph, the density of the edges in *G* is much higher and can be used to measure the edge density of the subgraph.

## Methods

Previous studies have shown that there are several strategies to upgrade the performance of the essential protein identification methods. The first one is to design novel centrality measures, which can provide crucial insights on the topological features of PINs. The second strategy is to denoise PINs to increase the precision of the interactions [29]. Another one is to identify essential proteins based on the fusion of other kinds of biological information or other kinds of identification methods.

**Fig. 2:**
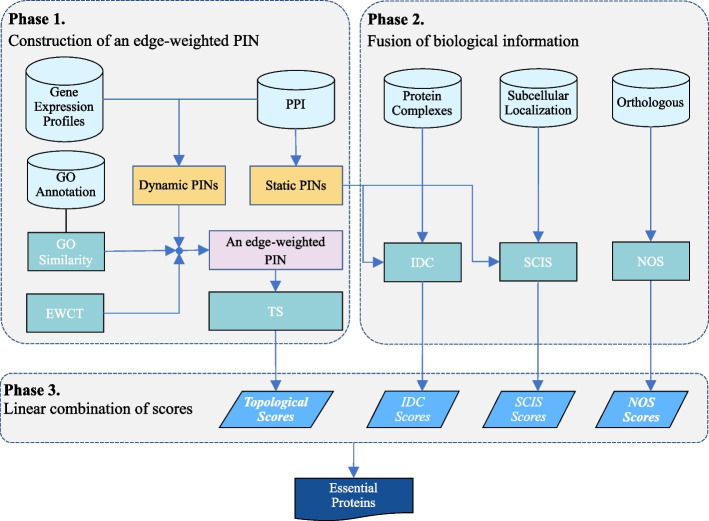
The framework of CTF

In this study, we present a new identification method based on a new centrality measure, DPINs, and the fusion of three kinds of biological information, namely, protein complex, subcellular location, and orthologous information, as shown in Fig. 2.

### Edge-weight function

There are four scores in the CTF method. The first one is a topological score computed based on an edge-weighted PIN. To construct an edge-weighted PIN, we first propose the EWCT function for the assignment of weights to edges.

The central idea of EWCT is to assign weights to the edges of PINs based on the edge features of the PINs and GO annotations. The topological features used in EWCT are *h*-quasi-cliques and *uv*-triangle graphs.

#### Theorem 1

Given a PIN $G_p = (V_p, E_p)$, for $(u, v) \in E_p$, let $C_1 = N(u) \cap N(v)$, and $C_2 = N(w_1) \cup N(w_2)$, where $w_1 \in {u, v}$ and $w_2 \in C_1$. Let $G_{uv} = (V_{uv}, E_{uv})$ be the induced subgraph on the vertex set ${u, v} \cup C_1 \cup C_2$. If $|V_{uv}| < 8$, then $G_{uv}$ is an *h*-quasi-clique, and it is also a *uv*-triangle graph.

#### Proof

We first show that $G_{uv}$ is an *h*-quasi-clique.

The number of edges in $G_{uv}$ is computed below. Let $n = |V_{uv}|$, $n_1 = |C_1|, n_2 = |C_2|, w \in {u, v}, v_1 \in C_1$, and $v_2 \in N(w) \cap N(v_1) \subseteq C_2$. Consequently, we have that $n = n_1 + n_2 + 2$. Observe that vertices *u*, *v*, and $v_1$ are vertices of a triangle in $G_p$, and the number of these triangles is $n_1$; vertices *w*, $v_1$, and $v_2$ are vertices of a triangle in $G_p$, and the number of these triangles is $n_2$. Therefore, the number of edges in Guv is at least $2n_1 + 2n_2 + 1 = 2n - 3$. The triangles formed by vertices *u*, *v*, and $v_1$ or *w*, $v_1$, and $v_2$ are triangle graphlets of $G_{uv}$.

In addition, for the clique $C_{uv} = (V_c, E_c)$ on the vertex set ${u, v} \cup C_1 \cup C_2$, we have $|E_c| = n(n - 1) / 2 = (n_1 + n_2 + 2)(n_1 + n_2 + 1) / 2$.

Since *n* is an integer and $0< n < 8$, Eq. (1) holds.

1 $$\begin{aligned} \frac{\left| E_{u v}\right| }{\left| E_c\right| }=\frac{2 n-3}{n(n-1) / 2}=\frac{2(2(n-1)-1)}{n(n-1)}=\frac{4}{n}-\frac{2}{n(n-1)} \ge \frac{4}{7}-\frac{2}{7 \times 6}>\frac{1}{2} \end{aligned}$$

Thus, $G_{uv}$ is an *h*-quasi-clique by Definition 1.

By the construction of $G_{uv}$ and Definition 2, we get that $G_{uv}$ is a *uv*-triangle graph. The theorem follows. $\square$

To the best of our knowledge, the average degree in a PIN is about 8, and the degrees of about 60–85% of proteins in a PIN are less than or equal to 7 such as shown in Table 1, in which there are 5 PINs, including Gavin, Krogan, DIP, MIPS, and MBD, for describing degree properties of vertices in PINs. We may conclude that the vertex number of a maximal clique in a PIN is lower than 8, and the vertex number of $G_{uv}$ is lower than 7 in most cases. The property of a PIN satisfies the conditions of Theorem 1 in most cases, that is, $G_{uv}$ is an *h*-quasi-clique and is also a *uv*-triangle graph.

**Table 1:** Degree properties of vertices in PINs

Datasets	Average vertex degree	Percentages of vertices (degree $\le$ 7)
Gavin	8.27	59.79
Krogan	7.80	71.10
DIP	9.72	68.00
MIPS	5.42	85.10
MBD	9.00	73.40

The important observation is that $G_{uv}$ is characterized by the richness of triangle graphlets. The edge feature of $G_{uv}$ can be used to compute the weight of (*u*, *v*).

To define the function EWCT, the two definitions below are used.

#### Definition 3

(Half of the Common Neighbors) For two vertices *u* and *v* in a PIN, the half of the common neighbors (HCN) of *u* and *v* is defined as Eq. (2).

2 $$\begin{aligned} \mid {\text {HCN}}(u, v) {\mid } =\frac{|N(u) \bigcap N(v)|}{2} \end{aligned}$$

#### Definition 4

(Summation of All Neighbor Supports) For two vertices *u* and *v* in a PIN, the summation of all neighbor supports (SANS) is the summation of the product of *HCN*(*u*, *w*) and *HCN*(*w*, *v*), where *w* is a common neighbor of *u* and *v*.

3 $$\begin{aligned} {\text {SANS}}(u, v)=\sum _{w \in (N(u) \cap N(v))}({\text {HCN}}(u, w) \times {\text {HCN}}(w, v)) \end{aligned}$$

Note that, as illustrated above, the vertex set $\{u, v\} \cup (N(u) \cap N(v)) \cup ((N(u) \cap N(w)) \cup (N(v) \cap N(w)))$ is an *h*-quasi-clique in most cases and is also a *uv*-triangle graph.

On the basis of HCN and SANS, we define the function EWCT by Eq. (4) used to compute the importance of edge $e = (u, v)$. In addition, GO annotations can be used to adjust the weights of the edges as stated above. We use the function *Go*(*v*, *u*) proposed by Wang [39] to adjust the edge weights, where the value of *Go*(*v*, *u*) is between 0 and 1.

For two vertices *u* and *v* in a PIN, the function EWCT is defined as Eq. (4), where the divisor in Eq. (4) is used to balance the difference of the neighbor numbers for different vertices.

4 $$\begin{aligned} {\text {EWCT}}(u, v)=\frac{{\text {SANS}}(u, v)-{\text {HCN}}(u, v)}{(|N(u) \cup N(v)|+1)} \times G o(u, v) \end{aligned}$$

The meaning of function EWCT(*u*, *v*) is that its value is highly correlated with two edge features *h*-quasi-cliques and *uv*-triangle graph.

**Fig. 3:**
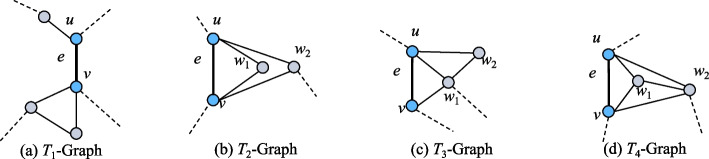
The four common kinds of graphlets in PINs

For example, previous studies have shown that the neighborhood topology of a PIN is highly correlated with the essentiality of proteins. Based on the neighborhood topology of a PIN, four kinds of subgraphs occur frequently in PINs as shown in Fig. 3, called $T_1$-Graph, $T_2$-Graph, $T_3$-Graph, and $T_4$-Graph, where the edge $e = (u, v)$ will be assigned a weight. As detailed in Fig. 3, the solid edges are the characterizing edges used to compute the weight of *e*. The features of these four graphlets are described in Table 2. If we only consider topological features by omitting GO annotations in Eq. (4), that is, set *Go*(*u*, *v*) to 1, the EWCT values of the edge e in $T_1$-Graph, $T_2$-Graph, $T_3$-Graph, and $T_4$-Graph are 0, 0, 0.2, and 1.2, respectively. That is, higher EWCT values lead to more important edges.

**Table 2:** The features of four graphlets

Graphlets	Features	Importance of edge e
$T_1$-Graph	$|N(u) \cap N(v)| = 0$, and there may only exist an edge between two vertices belonging to *N*(*w*), where $w \in \{u, v\}$.	Weak
$T_2$-Graph	$|N(u) \cap N(v)| \le 2$, and *u*, *v*, and there does not exist an edge between two vertices belonging to $N(u) \cap N(v)$.	Weak
$T_3$-Graph	$|N(u) \cap N(v)| = 1$, and there is an edge incident on *w*_1_ and *w*_2_, where $w_1 \in N(u) \cap N(v)$, $w_2 \in (N(u) \cup N(v) - (N(u) \cap N(v))$.	Medium
$T_4$-Graph	$|N(u) \cap N(v)| \ge 2$, and ${u, v, w_1, w_2}$ induces a clique.	Strong

Furthermore, we also analyze the computational complexity of the EWCT method. The basic operation of EWCT is to compute the common neighbor set of *u* and *v*, that is, $N(u) \cap N(v)$. Therefore, the computational complexity of EWCT is $O(d(u) \times \log (d(v))$. To compute the weights for all $e \in E$, the computational complexity is $O(|E| \times d(u) \times \log (d(v))$. As the average degree in a PIN is about 8, the EWCT function can be efficiently computed.

### Construction of an edge-weighted PIN

It is well known that PINs obtained through high-throughput methods have a high level of noise. This leads to difficulties in identifying essential proteins. A PIN is also called a static PIN to distinguish from a DPIN. In addition, interactions among proteins are dynamic in a cell, that is, a static PIN cannot reflect the dynamic feature of interactions.

To tackle these two problems, especially the noise in the form of false positives, we construct a DPIN by combining static PINs with gene expression profiles. This paper applies the 3-*sigma* method proposed by Wang et al. to construct DPINs [30].

A DPIN is defined as a 4-tuple $DG = (V, E, T, \text {ATE})$, where *V* and *E* correspond to proteins and interactions of PINs, respectively, $T = \{T_i | 1 \le i \le n\}$ is a set of active time points for proteins, and ATE is a function whose value is the active time attribute set of proteins. A snapshot of a DPIN is defined as a 3-tuple $DG_{i} = (V_i, E_i, \text {ATE}(u, v, T_i))$, where $V_i \in V$ and $E_i \in E$ are active at time point $T_i \in T$, $\text {ATE}(u, v, T_i)$ is used to compute the active probability of vertices *u* and *v* in $V_i$ at time point $T_i$, and $i \in [1,|T|]$.

Given a DPIN subnetwork $DG_i = (V_i, E_i, {\text {ATE}}(u, v, T_i))$, the weight of edge (*u*, *v*) is computed using the function $\text {EWD}(u, v, T_i)$ as Eq. (5). Recall that gene expression profiles are used to construct DPINs, and the gene expression profiles used in our experiments are 12 time intervals per cycle. Therefore, the number of active time points is 12 for a gene in a cycle, that is, $|T| = 12$.

5 $$\begin{aligned} {\text {EWD}}\left( u, v, T_i\right) ={\text {ATE}}\left( u, v, T_i\right) \times {\text {EWQC}}(u, v), T_i \in T, i \in [1,12] \end{aligned}$$

As detailed in Algorithm 1, the method CEP (construction of an edge-weighted PIN) is used to construct an edge-weighted PIN. CEP contains 12 iterations, and each iteration processes a DPIN subnetwork and consists of two major steps. To begin with, compute the EWD value by Eq. (5)), and after that, we delete the trivial edges.
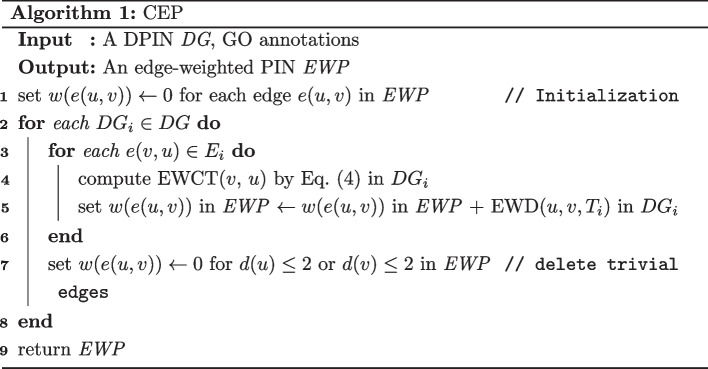


The interactions with high weights tend to connect essential proteins. After obtaining an edge-weighted PIN, it will be used to compute the topological score of a protein.

### Essentiality scores based on edge features

For protein *u* in an edge-weighted PIN, the topological score function defined by Eq. (6), named TS(*u*), is used to compute the topological score of *u* based on the weights of edges adjacent to *u*.

6 $$\begin{aligned} \textrm{TS}(u)=\sum _{v \in N(u)} \frac{w(e(u, v))}{2} \end{aligned}$$

**Fig. 4:**
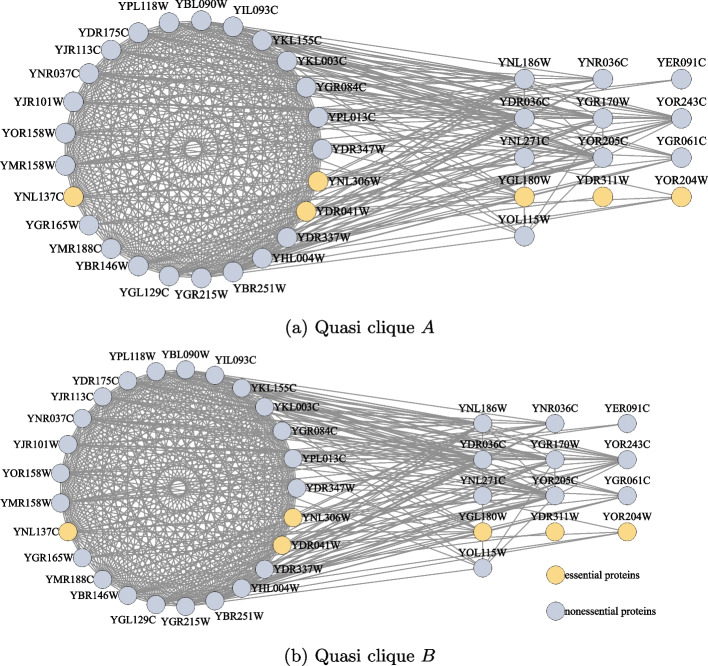
Non-essential proteins in a large quasi-clique

Normally, the range of TS(*u*) is from 0 to 100. Accordingly, if the value of TS(*u*) is too high, it is treated as an abnormal value. In fact, most of the proteins with too high topology scores are not essential, and their topology scores are assigned 0 by a threshold. In practice, we take 1000 as the threshold of TS(*u*). For example, as shown in Fig. 4a and b, respectively, there are 32 and 25 high-score proteins arranged in circles, whose scores are greater than 1000 in the Gavin dataset. The subgraph induced by these proteins is a quasi-clique. The quasi-clique with 1 essential protein has 32 vertices and 458 edges in Fig. 4a, and the quasi-clique with 3 essential proteins has 25 vertices and 289 edges in Fig. 4b. For the proteins arranged in a circle, their topology scores are set to zero.

### Essentiality scores based on biological information

As pointed out above, previous studies indicate that the use of biological information can improve the accuracy of essential protein identification. This paper applies three kinds of biological information, namely, protein complexes, subcellular localizations, and orthologous information.

A protein complex is a group of proteins that mutually interact, that is, protein complexes are substructures of a PIN. For a protein in a complex, the essentiality highly positively correlates with the participation degree [40].

Subcellular localization information is vital to understand the functions of proteins and is easily obtained. From a biological view, for two proteins, there is an interaction between them if and only if they are in the same subcellular compartment [41]. Subcellular localization information can be used to reduce the noise in PINs and is helpful for further improvement of identification accuracy.

Because orthologous proteins have evolved from a common ancestor, they often perform the same function. The SON method proposed by Li et al. applied orthologous information, subcellular localization, and PINs to identify essential proteins [36]. Some previous studies also showed that the identification accuracy of essential proteins could be improved using orthologous information.

Based on these reports, this paper identifies essential proteins by the fusion of three kinds of biological information mentioned above.

### CTF method

Comparisons of the essential protein sets identified by the methods TS, IDC, SCIS, and NOS, show that these methods are complementary. In this paper, we first compute essentiality scores of proteins by four scores, namely, the topology score TS and three kinds of biological information scores as shown in Eq. (7), where IDC(*u*), SCIS(*u*), and NOS(*u*) are obtained from protein complexes, subcellular localizations, and orthologous information, respectively. These four scores are combined via a linear combination. Then, we rank proteins by essential scores in descending order, and the higher-ranked proteins are more likely to be essential proteins, that is, we can choose the top *k* proteins as essential candidates.

7 $$\begin{aligned} \begin{aligned} {\text {CTF}}(u)= \alpha \times \left[ \alpha \times \left( \frac{{\text {TS}}(u)+{\text {IDC}}(u)}{2}\right) +(1-\alpha ) \times 100 \times {\text {NOS}}(u)\right] \\ +(1-\alpha ) \times {\text {SCIS}}(u) \end{aligned} \end{aligned}$$

Note that the value of NOS ranges from 0 to 1 in practice. By contrast, those of TS, IDC, and SCIS range from 0 to 100, that is, the value of NOS is much less than TS, IDC, and SCIS. Subsequently, the value of NOS is amplified 100-fold in Eq. (7) to scale the four scores.

The parameter $\alpha \in$ [0, 1] is used to tune the rate of the four components TS, IDC, NOS, and SCIS. If $\alpha$ is set to 1, the essential score is determined by TS and IDC, and if $\alpha$ is set to 0, the essential score is determined by NOS and SCIS. If $\alpha$ is between 0 and 1, essential scores are computed according to the percentages of TS, IDC, NOS, and SCIS. In CTF, $\alpha$ is set to 0.4, and the reason is described in Subsection “Parameter settings”.

The details of the CTF method are described in Algorithm 2.
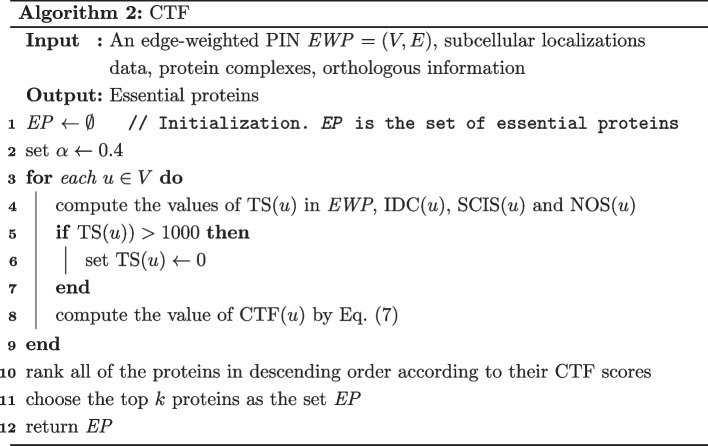


## Experiments and discussions

### Experimental data

In this study, multiple biological datasets from the baker’s yeast *Saccharomyces cerevisiae* are used, namely, PINs, GO annotations, gene expression profiles, subcellular localizations, protein complexes, orthologous information, and standard essential proteins. *Saccharomyces cerevisiae* has been widely used for essential protein studies because it is one of the most intensively studied organisms in molecular and cell biology, and it contains the most complete PPIs and rich biological information. Therefore, we evaluate the performance of CTF based on *Saccharomyces cerevisiae* datasets as shown in Table 3.

**Table 3:** Multiple biological datasets used for evaluating the performance of CTF

Data type	Data source	Quantity
PINs	DIP, Krogan, and Gavin	DIP: 24,743 interactions among 5093 proteins
		Krogan: 14,317 interactions among 3672 proteins
		Gavin: 7669 interactions among 1855 proteins
GO annotations	Saccharomyces GENOME Database (SGD)	42,878 GO annotations for 7014 proteins
Gene expression profiles	GEO (Gene Expression Omnibus), GSE3431 series	36 sample sites for 6777 gene expression sequences
Subcellular localizations	COMPARTMENTS Database	4865 proteins involved in 11 different localizations
Protein complexes	CM270, CM425, CYC408, and CYC428	745 protein complexes containing 2167 proteins
Orthologous information	InParanoid database	100 genomes (1 prokaryote and 99 eukaryotes)
Standard essential proteins	MIPS, SGD, DEG, and SGDP Database	1285 essential proteins, including 1167 in DIP, 929 in Krogan, and 714 in Gavin

### Comparisons with other methods

To show the advantage of our method CTF, three comparison methods are used, namely, statistical measures, top *k* proteins method, and receiver operating characteristic (ROC) and precision-recall (PR) curves.

#### Comparisons of statistical measures

For comparisons of CTF with some other existing algorithms, six statistical measures are employed, namely, sensitivity (*SN*), specificity (*SP*), positive predictive value (*PPV*), negative predictive value (*NPV*), *F*-measure (*F*), and accuracy (*ACC*). These measures are commonly used to measure the performance of essential protein identification. Let *TP* and *TN* denote the number of samples of the essential and non-essential proteins, which are identified correctly, respectively, and *FN* and *FP* denote the number of samples of the essential proteins and non-essential proteins, which are identified wrongly, respectively. These measures mentioned above are described as shown in Eqs. (8–13).

8 $$\begin{aligned} SN= \,& {} \frac{TP}{TP+FN} \end{aligned}$$

9 $$\begin{aligned} SP=\, & {} \frac{TN}{TN+FP} \end{aligned}$$

10 $$\begin{aligned} PPV=\, & {} \frac{TP}{TP+FP} \end{aligned}$$

11 $$\begin{aligned} NPV=\, & {} \frac{TN}{TN+FN} \end{aligned}$$

12 $$\begin{aligned} F= \,& {} \frac{2 \times SN \times PPV}{SN+PPV} \end{aligned}$$

13 $$\begin{aligned} ACC=\, & {} \frac{TP+TN}{TP+TN+FP+FN} \end{aligned}$$

According to previously published studies, about 20–30% of all proteins are essential in a PIN. Therefore, we choose the top 25% as essential proteins and the others as non-essential proteins. For CTF, the lowest scores of essential proteins are 21.36 in DIP (1167th), 21.2 in Krogan (929th), and 23.355 in Gavin (714th). The average of the lowest scores is 21.97 in three datasets. Therefore, we take 22 as the threshold for CTF.

If we only use the threshold to choose essential proteins, for some datasets, the size of the candidate set may be inappropriate. Therefore, the evaluation model of this paper is described as follows. Let *s* be the number of the essential candidates chosen by a threshold and *r* be 25% of the size of the dataset, then we choose the top $(s + r)/2$ as the essential candidates. Actually, experiment results show that the evaluation model is better than the simple threshold model or the top *k* model.

We compare CTF with 14 existing methods, including MON, JDC, and LBCC on the DIP, Krogan, and Gavin datasets. The results are shown in Tables 4, 5 and 6.

The comparison results show that CTF outperforms the other methods on DIP (Table 4) and Krogan (Table 5), and CTF outperforms other methods in terms of three measures, namely, *SN*, *NPV*, and *F*-measure on Gavin (Table 6). Therefore, the CTF method has better performance than the other existing methods.

**Table 4:** Comparison of statistical measures of CTF and other methods on the DIP dataset

	*SN*	*SP*	*PPV*	*NPV*	*F*	*ACC*
BC	0.3710	0.7860	0.3401	0.8079	0.3549	0.6909
NC	0.4670	0.8146	0.4281	0.8372	0.4467	0.7349
CoEWC	0.4653	0.8141	0.4266	0.8366	0.4451	0.7341
PeC	0.4225	0.8013	0.3873	0.8236	0.4041	0.7145
WDC	0.4893	0.8212	0.4485	0.8440	0.4680	0.7451
ION	0.5441	0.8372	0.4984	0.8607	0.5203	0.7701
LAC	0.4739	0.8164	0.4341	0.8392	0.4531	0.7379
LBCC	0.2464	0.7382	0.2160	0.7699	0.2302	0.6065
TEO	0.4919	0.8220	0.4509	0.8448	0.4705	0.7463
esPOS	0.5064	0.8260	0.4639	0.8492	0.4842	0.7528
TEGS	0.5176	0.8296	0.4745	0.8526	0.4951	0.7581
JDC	0.4859	0.8199	0.4451	0.8429	0.4646	0.7434
DSN	0.5287	0.8327	0.4843	0.8560	0.5055	0.7630
MON	0.5433	0.8370	0.4976	0.8604	0.5195	0.7697
CTF	**0.5458**	**0.8609**	**0.5385**	**0.8645**	**0.5421**	**0.7887**

Bold values indicate the best reults in contrast experiments

**Table 5:** Comparison of statistical measures of CTF and other methods on the Krogan dataset

	*SN*	*SP*	*PPV*	*NPV*	*F*	*ACC*
BC	0.3628	0.7882	0.3671	0.7850	0.3649	0.6806
NC	0.4273	0.8101	0.4325	0.8068	0.4299	0.7132
CoEWC	0.4306	0.8112	0.4357	0.8079	0.4331	0.7149
PeC	0.4263	0.8097	0.4314	0.8065	0.4288	0.7127
WDC	0.4607	0.8214	0.4662	0.8181	0.4635	0.7301
ION	0.5371	0.8472	0.5436	0.8439	0.5403	0.7688
LAC	0.4284	0.8104	0.4336	0.8072	0.4310	0.7138
LBCC	0.4639	0.8225	0.4695	0.8192	0.4667	0.7318
TEO	0.4510	0.8181	0.4564	0.8148	0.4537	0.7252
esPOS	0.4672	0.8236	0.4728	0.8203	0.4700	0.7334
TEGS	0.4833	0.8290	0.4891	0.8257	0.4862	0.7416
JDC	0.4553	0.8195	0.4608	0.8163	0.4580	0.7274
DSN	0.4952	0.8330	0.5011	0.8297	0.4981	0.7475
MON	0.5274	0.8440	0.5338	0.8406	0.5306	0.7639
CTF	**0.5447**	**0.8611**	**0.5705**	**0.8481**	**0.5573**	**0.7810**

Bold values indicate the best reults in contrast experiments

**Table 6:** Comparison of statistical measures of CTF and other methods on the Gavin dataset

	*SN*	*SP*	*PPV*	*NPV*	*F*	*ACC*
BC	0.2815	0.7695	0.4332	0.6312	0.3413	0.5817
NC	0.3796	0.8309	0.5841	0.6815	0.4601	0.6571
CoEWC	0.3880	0.8361	0.5970	0.6858	0.4703	0.6636
PeC	0.3922	0.8387	0.6034	0.6880	0.4754	0.6668
WDC	0.4076	0.8484	0.6272	0.6959	0.4941	0.6787
ION	0.4314	0.8633	0.6638	0.7081	0.5229	0.697
LAC	0.3824	0.8326	0.5884	0.6830	0.4635	0.6593
LBCC	0.3810	0.8317	0.5862	0.6822	0.4618	0.6582
TEO	0.4398	0.8685	0.6767	0.7124	0.5331	0.7035
esPOS	0.3978	0.8422	0.6121	0.6909	0.4822	0.6712
TEGS	0.4258	0.8598	0.6552	0.7052	0.5161	0.6927
JDC	0.2577	0.7546	0.3966	0.6190	0.3124	0.5633
DSN	0.4510	0.8755	0.6940	0.7182	0.5467	0.7121
MON	0.4720	**0.8887**	**0.7263**	0.7290	0.5722	**0.7283**
CTF	**0.5630**	0.7932	0.6301	**0.7436**	**0.5947**	0.7046

Bold values indicate the best reults in contrast experiments

#### Comparisons of top *k* proteins

Similar to most comparisons, we also carry out comparisons of the top *k* proteins between CTF and other methods. We first rank proteins by essential scores in descending order, then choose the top *k* proteins as essential candidates and determine how many of these are essential.

To evaluate the performance of CTF, we compare it with 16 methods, namely, NC, PeC, WDC, ION, CoEWC, LAC, GEG, SON, LBCC, TEO, esPOS, TEGS, JDC, DSN, MON, and GEGSO on the DIP, Krogan, and Gavin datasets. The results are listed in Table 7, Table 8, and Table 9, in which the number of essential proteins in the top *k*-ranked proteins is shown, where *k* is set to 100, 200, 300, 400, 500, and 600. The results show that CTF outperforms the other compared methods in more than half of all cases.

**Table 7:** Number of proteins accurately predicted by CTF and 16 other compared methods on the DIP dataset

TOP *k*	100	200	300	400	500	600
NC	55	126	182	230	279	309
PeC	75	138	200	247	286	328
WDC	70	132	188	246	298	340
ION	78	155	220	276	330	379
CoEWC	80	133	182	234	276	316
LAC	59	120	176	228	266	306
GEG	80	160	214	261	300	334
SON	81	153	224	282	340	389
LBCC	74	135	205	262	308	361
TEO	82	153	218	276	320	365
esPOS	85	155	211	268	320	362
TEGS	82	163	234	289	345	397
JDC	80	153	224	267	315	355
DSN	**92**	**179**	**248**	298	340	391
MON	90	173	244	306	358	411
GEGSO	86	172	245	**314**	370	**432**
CTF	^2*nd*^ 91	^4*th*^ 167	^4*th*^ 242	^1*st*^ **314**	^1*st*^ **374**	^2*nd*^ 422

Bold values indicate the best reults in contrast experiments

**Table 8:** Number of proteins accurately predicted by CTF and 16 other compared methods on the Krogan dataset

TOP *k*	100	200	300	400	500	600
NC	66	131	184	220	272	305
PeC	80	137	183	221	261	300
WDC	72	136	199	242	274	315
ION	79	154	210	261	313	370
CoEWC	74	131	174	217	257	296
LAC	73	134	180	218	261	299
GEG	72	144	195	244	279	317
SON	84	158	215	275	329	374
LBCC	63	130	190	243	289	319
TEO	72	150	210	253	295	326
esPOS	72	131	189	236	272	315
TEGS	74	151	211	261	301	341
JDC	74	148	199	242	285	319
DSN	**91**	164	216	272	313	349
MON	88	**166**	232	292	343	390
GEGSO	81	156	217	280	335	381
CTF	^3*rd*^ 85	^4*th*^157	^1*st*^ **236**	^1*st*^ **307**	^1*st*^ **367**	^1*st*^ **408**

Bold values indicate the best reults in contrast experiments

**Table 9:** Number of proteins accurately predicted by CTF and 16 other compared methods on the Gavin dataset

TOP *k*	100	200	300	400	500	600
NC	33	106	175	232	293	349
PeC	44	125	201	249	292	325
WDC	41	119	195	252	311	356
ION	45	126	202	264	325	372
CoEWC	44	122	196	250	291	329
LAC	27	109	178	235	297	341
GEG	51	131	206	256	301	341
SON	82	166	235	295	336	382
LBCC	38	113	176	235	285	321
TEO	43	120	203	275	332	367
esPOS	38	112	181	254	298	354
TEGS	49	126	204	268	317	362
JDC	42	85	123	158	197	231
DSN	**95**	**174**	234	291	340	384
MON	92	170	**242**	**299**	**353**	**398**
GEGSO	48	130	205	277	324	387
CTF	^3*rd*^ 89	^4*th*^164	^4*th*^ 231	^2*nd*^ 296	^2*nd*^ 342	^2*nd*^ 389

Bold values indicate the best reults in contrast experiments

#### Comparison of ROC and PR curves

ROC and PR curves are commonly used to visually evaluate the performance of identification methods. A ROC curve is a graphical plot created by plotting the true positive rate (*TPR*, also called the sensitivity (*SN*), represented as Eq. (8)) against the false positive rate (*FPR*, represented as Eq. (14)), and a PR curve is a graphical plot created by plotting the *TPR* against the *PPV*.

14 $$\begin{aligned} FPR=\frac{FP}{FP+TN} \end{aligned}$$

As stated above, the proteins obtained by the methods are ranked by their scores in descending order. We choose the score of the *k*th protein as the threshold for CTF. The top *k* proteins are put into the positive set, which is the candidate set of essential proteins, and the others are put into the negative set, which is the candidate set of non-essential proteins, where $1 \le k \le 5093$ on the DIP data, $1 \le k \le 3672$ on the Krogan data, and $1 \le k \le 1855$ on the Gavin data. Then, the values of *TPR*, *FPR*, and *PPV* are calculated and plotted in the ROC and PR curves.

**Fig. 5:**
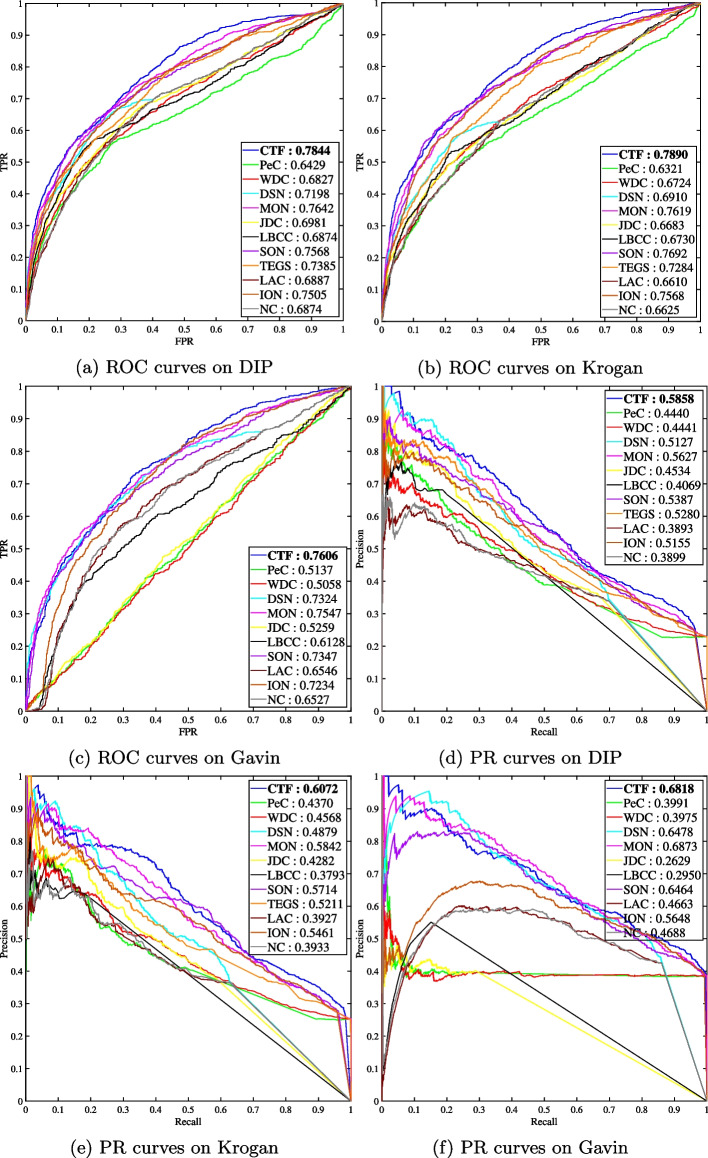
Comparison of AOC and PR curves of CTF and 11 methods based on DIP and Krogan and 10 methods based on Gavin

The area under the ROC or PR curve (AUC) is a measure used to evaluate the performance of identification methods. In general, a larger AUC value means better identification performance. The AUC values of ROC and PR for CTF and other existing methods are illustrated in Fig. 5.

Figure 5 indicates that CTF is very effective. In ROC analysis, CTF (blue) outperforms the other existing methods on three datasets as shown in Fig. 5a–c, and for PR analysis, CTF (blue) also outperforms the other existing methods on DIP and Krogan as shown in Fig. 5d and e. CTF has good performance on Gavin as shown in Fig. 5f. From the annotation numbers in Fig. 5, the values of AUC for CTF are significantly higher than the other existing methods.

### Ablation study

To elucidate the contributions of the CTF method, we perform an ablation study to investigate whether the EWCT-based measure TS and the usage of DPINs provide improvements in the identification performance. For investigating the effect of TS, we only use TS scores to identify essential proteins, and for investigating the effect of DPINs, we use static PINs instead of DPINs to compute the TS scores of the proteins.

#### Effect of the EWCT-based measure TS

To investigate the effects of the TS measure, we conduct an ablation study by removing three scores, namely, IDC, SCIS, and NOS, from CTF, that is, only use the topological scores computed by TS to identify the essential proteins and compare the results with other centrality measures, such as BC, SC, and LAC. The results in Tables 10 and 11 show that TS can identify more essential proteins than the other five centrality measures in most cases (83%) on static PINs and in all cases on DPINs. That is, TS outperforms other centrality measures, such as BC, SC, and LAC.

**Table 10:** Comparison of six centrality measures used to identify essential proteins on static PINs

		EWCT	DC	BC	CC	NC	LAC
DIP (TOP *k*)	100	^2*nd*^ 55	46	44	41	55	**59**
	200	^2*nd*^ 120	82	77	79	**126**	120
	300	^1*st*^ **182**	115	112	117	182	176
	400	^1*st*^ **235**	158	145	153	230	228
	500	^1*st*^ **286**	201	177	189	279	266
	600	^1*st*^ **333**	251	220	228	309	306
Krogan (TOP *k*)	100	^3*rd*^ 65	51	44	44	66	**73**
	200	^1*st*^ **134**	102	91	76	131	134
	300	^1*st*^ **184**	138	127	115	184	180
	400	^1*st*^ **235**	190	167	152	220	218
	500	^1*st*^ **277**	235	212	187	272	261
	600	^1*st*^ **314**	271	240	221	305	299
Gavin (TOP *k*)	100	^1*st*^ **76**	38	44	48	33	27
	200	^1*st*^ **152**	104	91	96	106	109
	300	^1*st*^ **217**	172	127	150	175	178
	400	^1*st*^ **281**	242	167	189	232	235
	500	^1*st*^ **336**	281	212	236	293	297
	600	^1*st*^ **375**	325	240	282	349	341

Bold values indicate the best reults in contrast experiments

**Table 11:** Comparison of six centrality measures used to identify essential proteins on DPINs

		EWCT	DC	BC	CC	NC	LAC
DIP (TOP *k*)	100	^1*st*^ **74**	43	43	30	55	71
	200	^1*st*^ **145**	93	78	60	123	127
	300	^1*st*^ **209**	140	103	92	184	181
	400	^1*st*^ **261**	178	140	119	236	223
	500	^1*st*^ **308**	223	177	144	279	267
	600	^1*st*^ **357**	269	215	175	316	323
Krogan (TOP *k*)	100	^1*st*^ **70**	62	48	41	69	67
	200	^1*st*^ **138**	108	95	83	133	118
	300	^1*st*^ **192**	156	131	123	180	182
	400	^1*st*^ **241**	202	177	156	229	231
	500	^1*st*^ **285**	246	210	181	267	272
	600	^1*st*^ **321**	281	248	224	310	311
Gavin (TOP *k*)	100	^1*st*^ **79**	36	43	49	25	28
	200	^1*st*^ **156**	102	92	96	92	95
	300	^1*st*^ **222**	165	132	151	157	165
	400	^1*st*^ **293**	239	172	199	205	232
	500	^1*st*^ **336**	294	213	248	264	288

Bold values indicate the best reults in contrast experiments

**Fig. 6:**
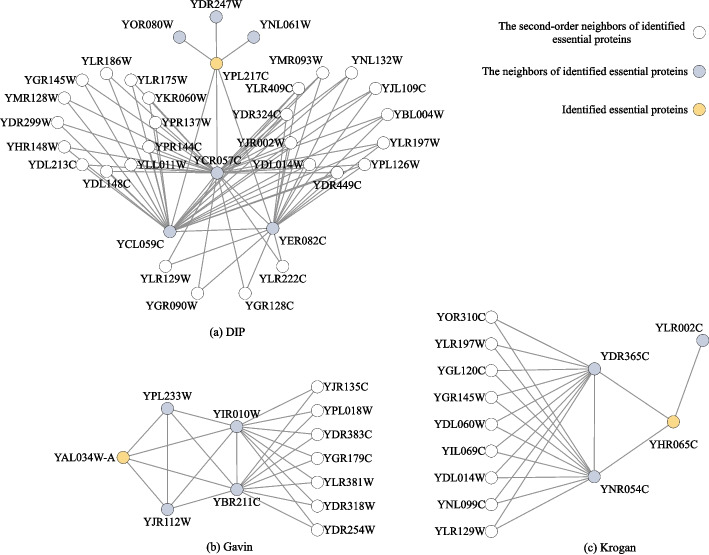
Essential proteins identified by TS and their neighbor structures while ignored by the other centrality measures

Further analysis indicates that there are some proteins identified as essential proteins by the TS measure but non-essential proteins by other centrality measures, such as BC, SC, and LAC. The common feature of these proteins is that they have low connectivity (degrees), but rich triangle graphlets formed by their second-order nearest neighbors. For example, as shown in Fig. 6, the proteins YPL217C in DIP, YAL034W-A in Gavin, and YHR065C in Krogan are identified as essential by TS but non-essential by BC, SC, and LAC.

#### Effect of DPINs

To demonstrate the effect of DPINs on the performance of CTF, we constructed ablation experiments, which use DPINs and static PINs to identify essential proteins. As shown in Table 12, when using DPINs, CTF can identify more essential proteins than using static PINs, that is, the results show that DPINs play an important role in the performance of CTF.

**Table 12:** Ablation experiments on DPINs and static PINs

	DIP	Krogan	Gavin
DPIN	**637**	**506**	**402**
Static PIN	602	501	395

Bold values indicate the best reults in contrast experiments

### Parameter settings

To balance the weight of the different components in CTF for improving accuracy, a proportional parameter $\alpha \in (0.1, 0.9)$ is adopted. As shown in Table 13, the number of essential proteins in top *k* proteins is shown, where *k* is set to 100, 200, 300, 400, 500, and 600 on the three datasets, and $\alpha$ is set to 0.1, 0.2, 0.3, 0.4, 0.5, 0.6, 0.7, 0.8, and 0.9. The highest number of essential proteins is shown in bold in Table 13 in each case. From the numbers in Table 13, we find that the best performance of CTF is achieved when $\alpha$ is set to 0.4.

**Table 13:** Effects of different $\alpha$ values, $\alpha \in (0.1 \text {--} 0.9)$

$\alpha$		0.1	0.2	0.3	**0.4**	0.5	0.6	0.7	0.8	0.9
DIP (TOP *k*)	100	72	81	87	91	**92**	89	87	86	83
	200	139	156	165	167	**172**	**172**	170	163	159
	300	209	227	239	242	242	243	**245**	238	234
	400	275	301	308	**314**	311	309	305	301	301
	500	335	360	372	**374**	**374**	371	360	353	346
	600	392	412	422	422	**425**	423	415	407	391
Krogan (TOP *k*)	100	75	83	**85**	**85**	81	78	79	76	75
	200	146	161	**166**	157	155	155	151	146	144
	300	218	236	**241**	236	235	228	222	216	212
	400	279	303	**311**	307	304	290	281	272	261
	500	337	352	362	**367**	359	351	343	324	307
	600	387	394	396	408	**412**	411	402	383	358
Gavin (TOP *k*)	100	78	81	86	**89**	86	78	65	55	48
	200	146	160	163	**164**	155	134	131	128	124
	300	222	225	**231**	**231**	213	213	210	206	202
	400	282	294	294	**296**	277	279	279	275	264
	500	334	**344**	**344**	342	339	333	332	327	326
	600	369	380	387	**389**	383	**389**	388	375	377

Bold values indicate the best reults in contrast experiments

## Conclusion

Essential proteins are very important for living organism survival, disease diagnosis and treatment, and drug design. The massively increasing number of PINs has enabled us to identify essential proteins using computing methods. To further improve the accuracy of identification, better centrality measures and the fusion of biological information are two crucial techniques.

In this paper, we presented the CTF method, based on *h*-quasi-cliques, *uv*-triangle graphs, and the fusion of three kinds of biological information. CTF first constructs an edge-weighted PIN to compute the topological scores of proteins and then computes the other three essential scores on the basis of three kinds of biological information. The analysis and experiments indicate that CTF has the following advantages. First, our method proposes the EWCT function for constructing an edge-weighted PIN used to compute the topological scores of proteins based on *h*-quasi-cliques, *uv*-triangle graph, and GO annotations. EWCT provides a deep insight into the inherent topological features of essential proteins. Second, to reduce the noise in PINs, CTF constructs an edge-weighted PIN using DPINs. In addition, CTF further upgrades the accuracy of identification through the fusion of three kinds of biological information. The experiment results on three PIN datasets show that CTF has substantially higher performance in terms of six statistical measures, including sensitivity, specificity, and *F*-measure, than other existing methods.

A well-defined centrality measure based on the topological features of PINs is still a very important issue, and to denoise PINs is another important issue. In future work, we plan to design better centrality measures and denoise PINs for identifying essential proteins.

## Data Availability

The Datasets used in this study, including PINs, GO annotations, gene expression profiles, subcellular localizations, protein complexes, orthologous information, and standard essential proteins, are from the public databases. The source code of the CTF method can be made available upon request from the corresponding author.

## Abbreviations

CEP: Construction of an edge-weighted PIN
CTF: Identification method of essential proteins based on edge features including *h*-quasi-Cliques and *uv*-triangle graphs, and the Fusion of multiple-source biological information
DPIN: Dynamic PIN
EWCT: Edge weight function based on edge features *h*-quasi-Cliques and *uv*-triangle graphs by combining with GO annotations.
HCN: Half of the common neighbors
SANS: Summation of all neighbor supports
TS($\cdot$): Topological score function
